# Relation of Trihalomethane Concentrations in Public Water Supplies to Stillbirth and Birth Weight in Three Water Regions in England

**DOI:** 10.1289/ehp.7111

**Published:** 2004-10-21

**Authors:** Mireille B. Toledano, Mark J. Nieuwenhuijsen, Nicky Best, Heather Whitaker, Peter Hambly, Cornelis de Hoogh, John Fawell, Lars Jarup, Paul Elliott

**Affiliations:** ^1^Small Area Health Statistics Unit, Department of Epidemiology and Public Health, Faculty of Medicine, and; ^2^Department of Environmental Science and Technology, Imperial College London, London, United Kingdom; ^3^Department of Statistics, Open University, Milton Keynes, United Kingdom; ^4^Independent Consultant, Buckinghamshire, United Kingdom

**Keywords:** chemical, disinfection, infant low birth weight, pregnancy outcome, stillbirth, trihalomethanes, water pollution, water purification

## Abstract

We investigated the association between total trihalomethanes (TTHMs) and risk of stillbirth and low and very low birth weight in three water regions in England, 1992–1998; associations with individual trihalomethanes (THMs) were also examined. Modeled estimates of quarterly TTHM concentrations in water zones, categorized as low (< 30 μg/L), medium (30–59 μg/L), or high (≥60 μg/L), were linked to approximately 1 million routine birth and stillbirth records using maternal residence at time of birth. In one region, where there was a positive socioeconomic deprivation gradient across exposure categories, there was also a positive, significant association of TTHM with risk of stillbirth and low and very low birth weight. Overall summary estimates across the three regions using a random-effects model to allow for between-region heterogeneity in exposure effects showed small excess risks in areas with high TTHM concentrations for stillbirths [odds ratio (OR) = 1.11; 95% confidence interval (CI), 1.00–1.23), low birth weight (OR = 1.09; 95% CI, 0.93–1.27), and very low birth weight (OR = 1.05; 95% CI, 0.82–1.34). Among the individual THMs, chloroform showed a similar pattern of risk as TTHM, but no association was found with concentrations of bromodichloromethane or total brominated THMs. Our findings overall suggest a significant association of stillbirths with maternal residence in areas with high TTHM exposure. Further work is needed looking at cause-specific stillbirths and effects of other disinfection by-products and to help differentiate between alternative (noncausal) explanations and those that may derive from the water supply.

Chlorination has been the main means for disinfecting municipal drinking water in many countries, including the United Kingdom, for many decades. The added chlorine reacts with naturally occurring organic matter to form a wide range of unwanted halogenated organic compounds, often referred to as disinfection by-products (DBPs). Among the most widely occurring by-products are trihalomethanes (THMs), haloacetic acids (HAAs), haloacetonitriles, and haloketones (Krasner et al. 1989; Nieuwenhuijsen et al. 2000b). Besides organic matter and chlorine dose, factors affecting the composition and concentration of DBPs include residence time in the distribution system, temperature, pH, and bromide levels (Chen and Weisel 1998; Krasner et al. 1989, 1994; Singer 1999; Stevens et al. 1989).

Of the DBPs, a group of four THMs [chloroform, bromodichloromethane (BDCM), dibromochloromethane (DBCM), and bromoform] generally occur at the highest concentrations in drinking water and are the DBPs for which standards are most commonly set. In consequence, they are routinely measured throughout water supplies and have been used as the exposure index in various epidemiologic studies that have examined the relationship between DBPs and adverse birth outcomes. These studies have, however, used a variety of study designs and methods of exposure assessment, and findings to date have been inconsistent (Nieuwenhuijsen et al. 2000a). Although some have reported significant excess risks of low birth weight or small size for gestational age (Bove et al. 1995; Gallagher et al. 1998; Kallen and Robert 2000; Kramer et al. 1992; Wright et al. 2003), others have not (Dodds et al. 1999; Jaakkola et al. 2001; Kanitz et al. 1996; Savitz et al. 1995; Yang et al. 2000). A number of studies conducted in Canada have found significant excess risks of stillbirths with higher total THM concentrations (Dodds et al. 1999, 2004) and chloroform and BDCM (King et al. 2000), whereas studies in other countries have found no excess risk (Aschengrau et al. 1993; Bove et al. 1995; Jaakkola et al. 2003). Evidence for an association with spontaneous abortion is sparse; two studies have reported significant excess risks (80–100%) (Aschengrau et al. 1989; Waller et al. 1998, 2001), whereas Savitz et al. (1995) found a smaller (20%), nonsignificant excess risk. Of the 10 studies to date that have examined various congenital anomalies, eight have shown excess risks for some of the congenital defects (Aschengrau et al. 1993; Bove et al. 1995; Cedergren et al. 2002; Dodds and King 2001; Dodds et al. 1999; Hwang et al. 2002; Klotz and Pyrch 1999; Magnus et al. 1999), particularly for all defects (Aschengrau et al. 1993; Bove et al. 1995; Hwang et al. 2002; Magnus et al. 1999), neural tube defects (Bove et al. 1995; Dodds and King 2001; Dodds et al. 1999; Klotz and Pyrch 1999; Magnus et al. 1999), and urinary defects (Aschengrau et al. 1993; Hwang et al. 2002; Magnus et al. 1999).

Despite these inconsistencies, the large number of people exposed to chlorinated water supplies means that potentially the population-attributable risk is high, even though the available evidence suggests that the risk, if any, for stillbirth and low birth weight in relation to THMs is small. Here we present the results of the first U.K. study to examine this question.

## Materials and Methods

**Figure 1:**
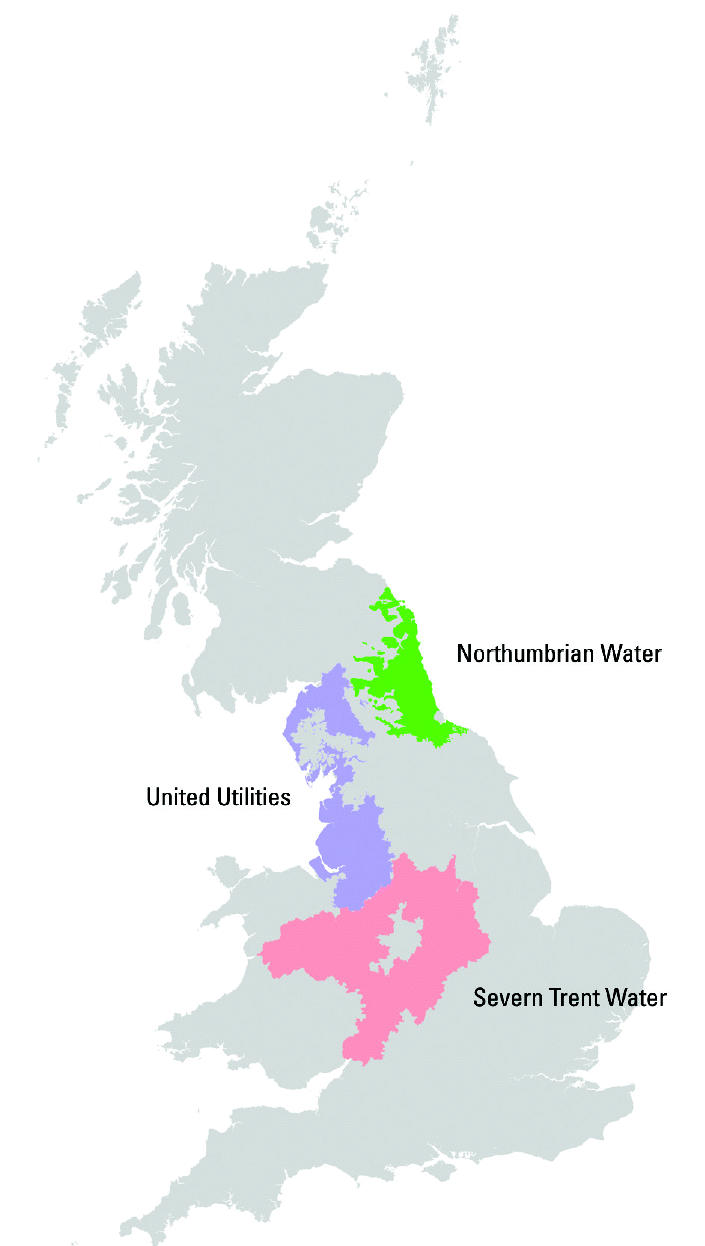
Locations of the study water company supply regions in Great Britain.

Public water supplies in the United Kingdom are statutorily divided into water zones, each zone covering a population of < 50,000. Less than 1% of households in the United Kingdom have private water supplies. The study was carried out in regions covered by three water companies in the north and midlands of England: Northumbrian, United Utilities (formerly North West), and Severn Trent (Figure 1), for which published data on THMs showed a wide range of exposure variation across water zones, and data on water zone boundaries were available. Northumbrian Water supplies approximately 2.6 million people across 120 water zones; United Utilities Water, 6.8 million people across 315 water zones; and Severn Trent Water, 7.4 million people across 300 water zones.

Where available, digital boundaries of water zones and their identification codes were obtained from each water company for each year under study. Alternatively, paper maps providing such details were obtained and digitized in-house, using the ArcInfo (version 7.02; Environmental Systems Research Institute, Redlands, CA, USA) geographical information system (GIS). Boundary data were available for the following years: Northumbrian, 1997; United Utilities, 1992–1997, and Severn Trent, 1993–1998.

Individual postal-coded records were extracted from the national birth and stillbirth registers held at the U.K. Small Area Health Statistics Unit (SAHSU; London, UK). Low birth weight was defined as < 2,500 g, and very low birth weight as < 1,500 g. Registration of all stillbirths is a legal requirement in the United Kingdom, providing a national register with high levels of ascertainment. Since the end of 1992, stillbirths are legally defined as fetal deaths after 24 completed weeks of gestation. Birth and stillbirth records were subsequently linked to data on water supply for the three regions. A link between postal code and water zone was created using point-in-polygon methods within the GIS software to allocate each postal code to its water supply zone. Postal code locations were derived from the historical postal code file for Great Britain, developed by SAHSU. This file traces postal codes back in time and assigns a grid coordinate for each postal code in each year. To take account of changes in the location of both postal codes and water zone boundaries over time, a separate link was created for each year of the study period.

### Exposure data.

We used THM concentrations as the marker for chlorination byproducts in this study. Water samples are routinely collected and analyzed from each water zone using random samples at the tap. Under the regulations operating during the study period, the standard sampling frequency for THMs was a minimum of four samples per annum. However, if there was a breach of the standard of 100 μg/L for total THMs (TTHMs) as a rolling 3-month average (or, where samples were too few, a maximum concentration > 100 μg/L in any one sample), the sampling frequency increased to a minimum of 12 or 24 per annum depending on the zone size. Conversely, if the TTHM concentration was consistently < 50% of the standard, a reduced frequency of a minimum of one per annum could be used. The number of THM samples that had been collected and recorded in each zone was highly variable, ranging from 1 to 80 measurements in a year (Whitaker et al. 2004). The mean number of samples per region, per year, was 11.2 for United Utilities Water, 6.3 for Severn Trent Water, and 4.5 for Northumbrian Water. In addition, a number of THM measurements were below the limit of detection, with percentages ranging across regions as follows: chloroform, 3.5% (United Utilities) to 23% (Severn Trent); BDCM, 4.9% (United Utilities) to 22% (Severn Trent); DBCM, 16% (Severn Trent) to 82% (Northumbrian); and bromoform, 21% (Severn Trent) to 85% (United Utilities) (Whitaker et al. 2003b).

Because of the small number of THM measurements in some water zones, the need for quarterly (3 months) estimates (to allow for trimester weighted exposure estimates), and the problem of measurements below the limit of detection, it was necessary to model the raw THM data to obtain more robust estimates of the mean TTHM concentration in each zone. This was done using a hierarchical mixture model in the software WinBUGS (Bayesian inference using Gibbs sampling) (Spiegelhalter et al. 1996), as described elsewhere (Whitaker et al. 2004). Briefly, modeling was carried out separately for each water company and year. In each case, the data were transformed to approximate normality using an appropriate Box–Cox transformation (Box and Cox 1964). The model calculated the mean annual individual THM concentrations for each water zone and subsequently assigned an estimated water source type to each water zone depending on the four THM levels within each zone. We fitted a three-component mixture model in which zones were assumed to belong to one or some mixture of three components that we labeled “ground,” “lowland surface,” and “upland surface” waters (the components may not strictly correspond to these three water source types; we simply aimed to group waters with similar THM profiles, which are more likely to be shared among water of the same source type). Constraints were imposed on the model such that, for example, on average, chloroform concentrations were highest in the two “surface water” components and bromoform concentrations were highest in the “groundwater” component. These constraints were based on *a priori* knowledge about the relative concentrations of different THMs in different water sources, and were necessary to identify the three components in the mixture model. The hierarchical model was assigned over the zone-specific mean individual THM concentrations, enabling zones to “borrow” information from other zones with the same water source type. This resulted in more stable estimates for zones where few samples were taken. For measurements under the detection limit, we modeled to obtain an estimate between zero and the detection limit, rather than arbitrarily assigning half or two-thirds the detection limit, which is common practice. Seasonal variation was taken into account by estimating a quarterly effect common to all zones supplied by the same source type. These quarterly zone mean THM estimates were then back-transformed onto the original scale and summed to give TTHM levels.

The postal code of the maternal residence at the year of birth was used to identify the water zone of interest and hence the appropriate exposure status for each birth record. Because the final trimester may be the most relevant trimester of pregnancy for both low birth weight and stillbirth (Kline et al. 1989; Pless 1994), we obtained exposure status by calculating a weighted average of the modeled quarterly TTHM estimates for the appropriate zone for the last 93 days before the date of birth. The weighting was based on the proportion of the trimester falling into each quarterly period. Because data on gestation weeks at birth were unavailable, we were unable to allow for pregnancies that had not gone to term. For full-term pregnancies, the last 93 days would equate to the third trimester. For a premature fetus, this period would be the last 93 days of the pregnancy, which will include part of the second trimester. Births occurring in the first 93 days of the first year of the study for each company were excluded. Finally, the weighted average TTHM estimate associated with each birth record was categorized into one of three predefined exposure categories: low (< 30 μg/L), medium (30–59 μg/L), or high (≥60 μg/L). These were chosen with reference to the published literature on the possible associations of birth outcomes with TTHMs (Nieuwenhuijsen et al. 2000a) and with regard to the distribution of TTHM levels across the three water regions.

### Study population size.

The study population comprised all births in the water regions for a varying number of years between 1992 and 1998. A total of six (0.02%), 3,471 (0.68%), and 24,157 (4.5%) births were excluded in the Northumbrian, United Utilities, and Severn Trent areas, respectively. Reasons for exclusion included births occurring in water zones that could not be assigned an exposure estimate because of zone code-boundary mismatches; postal codes of births falling into gaps or overlaps between different water zone boundaries; lack of water zone identification provided with the zone boundary data; or water supplied to that postal code not from one of the three study water companies but from another water company or a private water supply. There were no material differences in the birth weight and stillbirth profiles of these excluded births and those that were retained in the study. A further 592 (2.9%), 12,247 (2.5%), and 13,888 (2.8%) multiple births were excluded in the three regions, respectively. This left 20,624 total (live and still) births in Northumbrian in 1997, 412,973 in United Utilities in 1993–1997 (data for 1992 were omitted because of a national change in the definition of a stillbirth), and 486,974 in Severn Trent for 1993–1998. Analysis of birth weight was restricted to live-birth records with a birth weight > 200 g (99% of birth weights < 200 g were recorded as zero), giving, for these analyses, 20,452 live births in Northumbrian in 1997, 467,597 live births in United Utilities in 1992–1997, and 481,255 in Severn Trent in 1993–1998.

### Statistical methods.

Using the statistical package S-Plus (Insightful, Seattle, WA, USA), we performed descriptive analysis for all three outcomes—stillbirth, low birth weight, and very low birth weight—in each region separately, as well as univariate and multiple logistic regression modeling with adjustment for measured potential confounders. Sex and maternal age (for which individual-level information was available) were considered as potential confounders, as was socioeconomic deprivation measured at the small-area level, according to location of the postal code of maternal residence at the time of birth. Maternal age was represented in five categories: ≤ 20, 21–25, 26–30, 31–35, and ≥ 36 years. Deprivation was measured by quintiles of the Carstairs index (Carstairs and Morris 1991), a combination of four indicators from the 1991 census at the level of enumeration district (the smallest geographic area for which British census data are available, with, on average, 400 people): the percentage of people with no car, percentage living in overcrowded housing, percentage with the head of household in social class IV (partly skilled occupations) or V (unskilled occupations), and the percentage of men unemployed. In the regression models, only potential confounders that led to a significant (*p* < 0.05) change in the model deviance or led to > 5% change in the log odds ratio (OR), in at least one of the three study regions, were included in the final models (Greenland and Rothman 1998a). Interaction parameters between THM category and all other covariates were tested in the final models.

Generalized additive models were fitted using smoothing splines (Hastie and Tibshirani 1990), to examine the shape of association, and to check linearity assumptions, for both continuous THM estimates and Carstairs deprivation score in each water region separately. In addition, a multilevel model with random water zone effects was fitted using the glmmPQL function in S-Plus (version 6.2, function available from Modern Applied Statistics with S-Plus library; Insightful) (Leyland and Goldstein 2001; Venables and Ripley 2002) to check for residual clustering of the outcomes within water zones. Finally, tests for heterogeneity of the ORs associated with THM exposure across the three water regions were performed and a random-effects model was used to obtain an overall summary estimate of the effect of THM allowing for heterogeneity in the region-specific estimates (Dersimonian and Laird 1986). All analyses were carried out for TTHMs, chloroform, BDCM, and total brominated THMs (the sum of BDCM, DBCM, and bromoform). (Levels of DBCM and bromoform were often below the detection limit and too low for categorization and meaningful analysis in the three regions under study.)

## Results

### Descriptive analysis.

**Figure 2:**
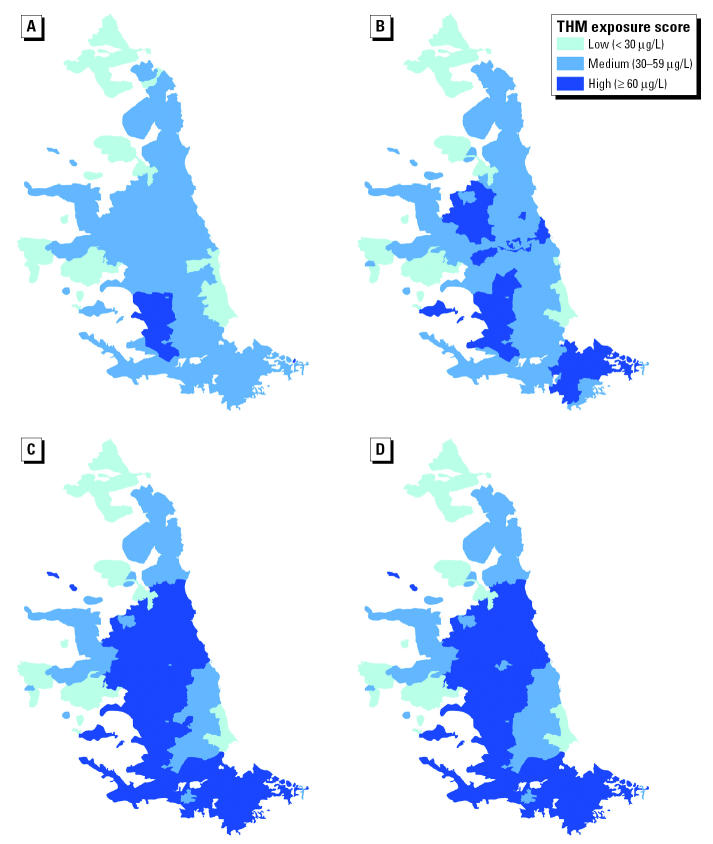
Maps showing water-supply-zone-level TTHM exposure categories for each quarter: Northumbrian Water, 1997: (*A*) January–March; (*B*) April–June; (*C*) July–September; (*D*) October–December.

**Figure 3:**
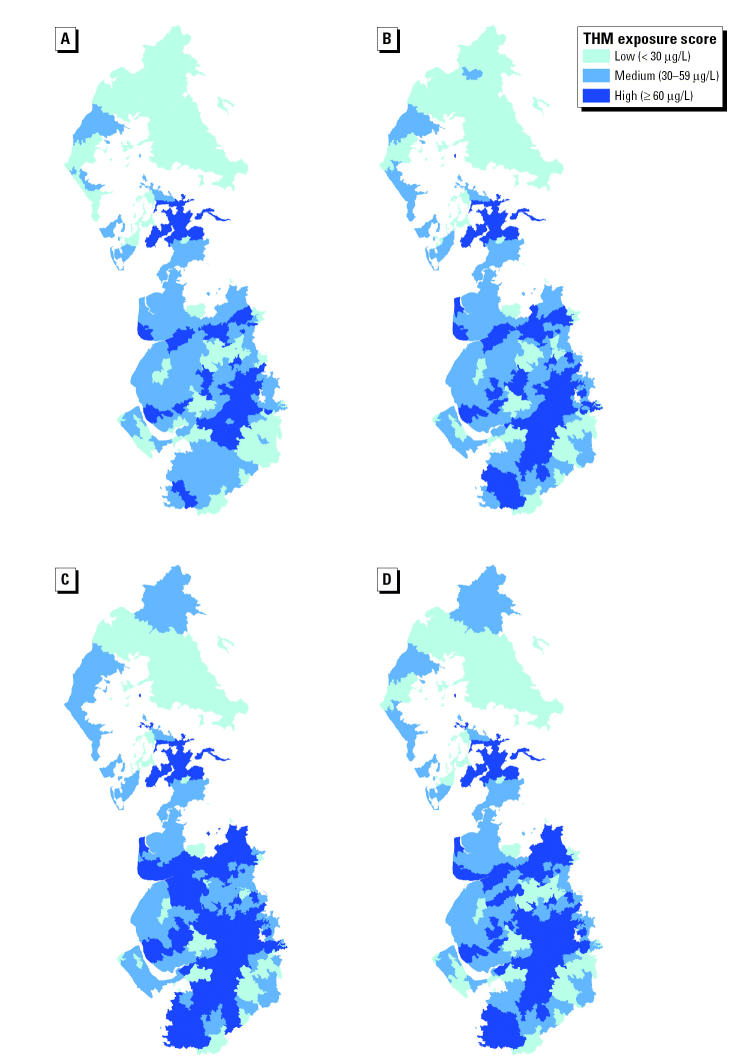
Maps showing water supply-zone-level TTHM exposure categories for each quarter, United Utilities Water, 1997: (*A*) January–March; (*B*) April–June; (*C*) July–September; (*D*) October–December.

**Figure 4:**
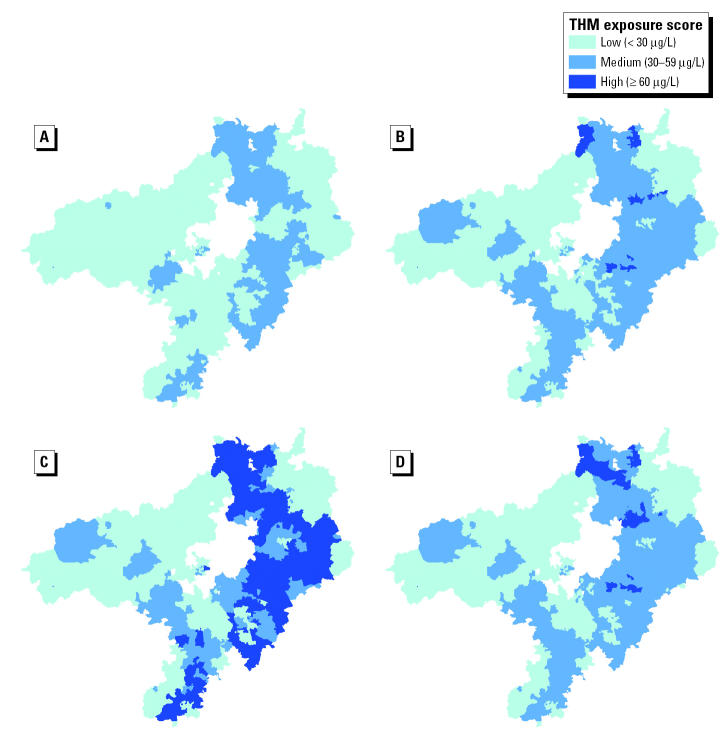
Maps showing water supply-zone-level TTHM exposure categories for each quarter, Severn Trent Water, 1997: (*A*) January–March; (*B*) April–June; (*C*) July–September; (*D*) October–December.

Descriptive data are shown in Table 1. Mean prevalence across the three regions ranged from 5.2 to 5.4 per 1,000 live and stillbirths for stillbirth, and from 61.5 to 64.8 and 9.1 to 10.7 per 1,000 live births for low and very low birth weight, respectively, whereas mean birth weight ranged from 3,337 to 3,351 g. Northumbrian was the most deprived region, and Severn Trent was the most affluent (mean Carstairs scores of 1.54 and 0.65, respectively). Maps for each region showing TTHM exposure classification by water zone and quarter are shown in Figures 2–4.

Average TTHM concentrations were similar for Northumbrian and United Utilities (56.6 and 52.0 μg/L, respectively), whereas the average concentration in Severn Trent was somewhat lower (35.8 μg/L); the average concentration in each of the three exposure categories was, however, similar in all three regions (Table 1). A tendency for increasing deprivation across the exposure categories (low to high) was seen in United Utilities but not in the other two regions. A pattern of higher rates of stillbirth and low and very low birth weight and lower mean birth weight was also seen across increasing exposure categories in the United Utilities region. In Severn Trent, there was a tendency for the reverse pattern for low and very low birth weight but not for stillbirths.

### Regression models.

Univariate logistic regression analysis for stillbirths and low and very low birth weight confirmed a trend of increasing prevalence with higher TTHM concentrations in United Utilities but not in the other regions. In United Utilities, the unadjusted ORs [95% confidence intervals (CIs)] for stillbirth in medium versus low and high versus low-exposure categories were 1.21 (1.04–1.41) and 1.34 (1.15–1.57), respectively; for low birth weight they were 1.20 (1.15–1.25) and 1.37 (1.31–1.43), respectively; for very low birth weight, they were 1.15 (1.03–1.28) and 1.32 (1.18–1.48), respectively.

Table 2 shows the results of the multiple logistic regression analysis for stillbirths and low and very low birth weight for each water region after adjusting for potential confounders. Again, in the United Utilities region, the risk was always highest and significant in the high-exposure category, with intermediate risk in the medium-exposure category. In Severn Trent, no statistically significant association was found between risk of stillbirths and low birth weight and TTHM concentrations, although for very low birth weight the risk in the high-TTHM areas was lower than in the low-TTHM areas (OR = 0.90; 95% CI, 0.82–0.99). Nonsignificant excess risks in medium- and high-TTHM areas relative to low-TTHM areas were found in Northumbrian for each birth outcome; CIs were wide, reflecting the much smaller numbers of births included in this region. No significant interactions between TTHM exposure and any of the potential confounders were found in the multivariate analysis.

The ORs associated with TTHM exposure showed statistically significant heterogeneity between water regions, for both low and very low birth weight but not for stillbirths (Table 2, notes). Allowing for this heterogeneity using a random-effects model to obtain overall summary estimates of the TTHM effects, small excess risks were found in the high- compared with low-exposure areas for stillbirths and low and very low birth weight of 11% (95% CI, 0–23%), 9% (95% CI, ^–^7–27%), and 5% (95% CI, ^–^18–34%), respectively, with intermediate risks in the medium-exposure areas (Table 2). Only results for stillbirths were statistically significant.

Among the individual THMs, chloroform showed a similar pattern of risk for stillbirths and low and very low birth weight to that of TTHM, both for the overall summary estimates across the three regions and in each individual region. Concentrations of BDCM and total brominated THMs did not show any association with risk of stillbirths or low or very low birth weight (data not shown).

Analysis using smoothing splines showed that at concentrations up to approximately 80 μgL, the relationship between TTHM and each of the birth outcomes was consistent with linearity (at concentrations > 80 μg/L CIs were very wide, because this represented ≤5% of the births in each region) (plots not shown). Sensitivity analysis excluding births from wards where the proportion of ethnic minority groups was ≥20%, and use of empirical annual mean TTHM estimates did not materially alter the results (Toledano 2004). Similarly, multilevel modeling including random water zone effects had negligible impact on the regression coefficients and their standard errors (data not shown).

## Discussion

This is the largest study yet conducted of the association between DBPs in the public water supply, as measured by TTHMs, and stillbirth and birth weight. In the United Utilities region, we found a trend of increasing prevalence of low and very low birth weight and stillbirth from low- to medium- to high-exposure areas, but this was not apparent in the other regions. There was also a socioeconomic deprivation gradient across exposure categories in this region. There was strong evidence of heterogeneity between water regions in the effect of exposure to TTHMs for low and very low birth weight but not for stillbirths. A random-effects model was therefore used to obtain an overall summary estimate of the exposure effect because it allows for different biases and unmeasured factors in the different study regions and incorporates the heterogeneity of effects in the analysis of overall risk associated with TTHM. In the random-effects analysis, we found small but statistically significant excess risk in the high-TTHM exposure areas for stillbirths.

This study is approximately twice as large as all the previous studies combined on low birth weight (Bove et al. 1995; Dodds et al. 1999; Gallagher et al. 1998; Jaakkola et al. 2001; Kallen and Robert 2000; Kanitz et al. 1996; Kramer et al. 1992; Savitz et al. 1995; Wright et al. 2003) and four times the size of all other studies combined on stillbirths (Aschengrau et al. 1993; Bove et al. 1995; Dodds et al. 1999, 2004; Kallen and Robert 2000). Although one of the main strengths of this study is its size, this and its retrospective nature simultaneously limit the options available for exposure assessment. Clearly, it is not possible to obtain individual tap water samples at each maternal residence, or direct measures of individual exposure, in such a large-scale study, and there is an inevitable trade-off between specificity of the exposure assessment and study power. For these reasons, most studies have used an ecologic measure for exposure assessment (Aschengrau et al. 1993; Bove et al. 1995; Hwang et al. 2002; Jaakkola et al. 2001; Kallen and Robert 2000; Kanitz et al. 1996; Klotz and Pyrch 1999; Kramer et al. 1992; Wright et al. 2003; Yang et al. 2000); some, like us, have incorporated modeled ecologic exposure estimates to improve the exposure classification (Dodds and King 2001; Dodds et al. 1999; Gallagher et al. 1998; King et al. 2000), and only a few have obtained individual-level exposure information (Dodds et al. 2004; Savitz et al. 1995; Shaw et al. 2003; Waller et al. 1998, 2001). To the extent that all these approaches are bound to lead to exposure misclassification, varying degrees of error (both Berkson and classical error) (Nieuwenhuijsen et al. 2000b) will result, leading to loss of power and/or bias in estimates of exposure–disease associations, most likely (but not necessarily) toward the null (no effect).

In this study we used modeled ecologic quarterly estimates of TTHM concentrations for all birth locations in the study, taking into account THM profiles commonly associated with particular water sources and seasonal variation (Whitaker et al. 2004) to provide an improved and more robust exposure assessment. One particular advantage is that the exposures are estimated with comparable precision across all the zones and quarters because of the hierarchical links built into the model, which is important given the variable number of raw measurements available in different zones. Nonetheless, inevitably there will be a degree of exposure misclassification because all mothers in one water zone were assigned the same (ecologic) exposure estimate. No account was taken of the potential mobility of mothers during pregnancy and consumption of water outside the home, other activities affecting THM exposure such as swimming, and possible variability in THM concentrations within a water zone.

The possibility of exposure measurement error from residential mobility during pregnancy cannot be ruled out, because an American study found that > 20% of pregnant women moved residence between the time of conception and delivery (Shaw and Halinka 1991). Of course, if mothers move but remain within the same water supply zone, this should not introduce substantial measurement error unless within-zone variability is greater than between-zone variability (which is not the case for our data). Mobility from zone to zone could also result from the home and workplace being in different water zones. However, recent research on tap-water–related activities among pregnant women in the United Kingdom suggests that possible consumption of water outside the home is unlikely to be a major source of exposure misclassification (Kaur et al. 2004). For example, on average, only 18% of total fluid ingestion by study participants was cold tap water, and only 30% of this tap water was consumed outside the home (Kaur et al. 2004). Moreover, women drank almost equal amounts of cold tap water and bottled water at home, but at work and elsewhere they drank almost three times more bottled water than cold tap water (Kaur et al. 2004). The effects of variations in individual behaviors (e.g., ingestion, showering and bathing habits) on actual THM uptake, and their implications for this epidemiologic study, have been explored in a simulation study. This showed that a moderate to strong correlation (~ 0.6–0.8) could be expected between concentrations of chloroform in tap water and actual uptake by pregnant women, even when there is no information on individual behavior (Whitaker et al. 2003a). Furthermore, analysis of THM data inone of our study water regions (United Utilities) showed that between-zone variation was consistently larger than within-zone variation for both chloroform and BDCM, the main THMs. This suggests that water zone means are a valid way of differentiating exposure to THMs between individuals (Keegan et al. 2001). Taken together, the above suggests that our methods provided a valid approach to estimating TTHM exposure of individuals for use in our epidemiologic study.

To date, total THMs have been the main focus of epidemiologic investigation. However, total THMs may not be a good marker of the individual THMs (e.g., brominated compounds) and other by-products (e.g., haloacetates) that have recently been implicated with respect to adverse birth outcomes (King et al. 2000; Klotz and Pyrch 1999; Swan and Waller 1998; Wright et al. 2003). For example, we found only a moderate correlation between total THMs and the various individual THMs (Keegan et al. 2001; Whitaker et al. 2003b). In our study of the individual THMs, we found an association with chloroform but not with the brominated compounds. Findings of our overall summary analyses reflected in particular trends in United Utilities region; although differences in results between our water regions might partly be accounted for by differing sociodemography, they might also have been caused by differing composition of the DBPs or the presence of other substances or factors that are strongly correlated with THMs in one region but not in the others.

An important issue is the extent to which our results might be explained by unmeasured or uncontrolled confounding. We had only limited data on potential confounders, and information on potentially important risk factors, such as maternal smoking habits and gestational age, was not available. Some previous studies have shown a much stronger association between TTHM exposure and low birth weight for term births only (Gallagher et al. 1998), whereas others have detected no consistent associations of low birth weight among all births or term births (Jaakkola et al. 2001; Wright et al. 2003). Others have observed an increased risk, in particular, of small size for gestational age with high TTHM exposure (Bove et al. 1995; Kramer et al. 1992; Wright et al. 2003). It is not yet clear, therefore, whether the underlying association between low birth weight and TTHM concentrations reflects a risk for babies born prematurely but of appropriate size for their gestational age or fetal growth retardation among babies born at term. Although some of the discrepancies between studies may have been due to differences in design, Wright et al. (2003) recently reported that confounding by gestational age had a substantial impact on the association between birth weight and TTHM concentrations. Unfortunately, we were unable to examine this in our study because data on gestational age are not included on the routine birth records.

The diverse etiologic routes to low birth weight might be a possible explanation for the observed heterogeneity in effect of TTHM on low and very low birth weight but not on stillbirths. For example, there could be differing proportions of small-for-gestational-age and low-birth-weight preterm births among the three study regions (e.g., reflecting differences in ethnic minority mix), with a stronger association of THM exposure with one of these pathways to becoming a low-birth-weight baby, but not the other.

Another possible explanation for the observed heterogeneity in effect of TTHM exposure on low and very low birth weight could relate to differences in baseline rates between the regions. Severn Trent and Northumbrian were found to have a higher prevalence of low and very low birth weight in the low-exposure areas than did United Utilities. If the effects of TTHM exposure are additive rather than multiplicative, this could lead to heterogeneity of relative effect measures such as ORs (Greenland and Rothman 1998b). However, this would not explain an apparent inverse association for very low birth weight in the high-exposure category seen in Severn Trent. The reasons for the different baseline rates across regions are unclear and merit further investigation.

We did have information on socioeconomic deprivation at small-area scale. In contrast to the other two regions, the high-exposure areas in the United Utilities region tended to be more deprived than the low-exposure areas. This was an unexpected finding. Both stillbirth and low birth weight are related to deprivation (higher rates among lower social classes) (Dummer et al. 2000; Nordstrom and Cnattingius 1996; Parker et al. 1994; Rodriguez et al. 1995). Comparison of ORs without adjustment for deprivation (Carstairs index) with those after adjustment in the United Utilities region showed that ORs were reduced by up to about one-half, suggesting the possibility of residual confounding. This was explored in more detail using data from the Health Survey for England (Erens and Primatesta 1997) on smoking habits and ethnicity for women of reproductive age living in the United Utilities region; these data showed that higher proportions of women of nonwhite origin (7.5 vs. 1.3%) and women who smoke (39.7 vs. 31.6%) resided in high- than in low-exposure areas. Analysis of data for London from the St. Mary’s Maternity Information System (Chapple 1997) showed increased relative risks (ranging from 1.4 to 3.2) among offspring of women who smoke and women who are of nonwhite origin, for each of the birth outcomes under study. Using these data, the higher proportion of nonwhite women living in areas of high compared with low TTHM concentrations in the United Utilities region would explain only around 13, 4, and 5% of the excess risk for stillbirth, low birth weight, and very low birth weight, respectively, whereas the higher proportion of women smokers living in areas of high compared with low TTHM concentrations would explain only around 3, 5, and 3%, respectively, of the excess risk (Toledano 2004). These excesses are generally less than or similar to the difference between the unadjusted and adjusted (for deprivation) risk estimates for each of the birth outcomes, suggesting that inclusion of the Carstairs index may have adequately adjusted for deprivation-related effects in the United Utilities region. Nonetheless, residual confounding by socioeconomic deprivation cannot be excluded. Excess risks in areas of high deprivation relative to areas of low deprivation (after adjustment for all other potential confounders and TTHM category) across the three water regions were, on average, 15–20 times the magnitude of those found in association with areas of high relative to low TTHM exposure, after adjustment for socioeconomic deprivation and other potential confounders.

If, however, our results reflect some causal association rather than confounding or other source of bias, what could be the potential mechanisms? The THMs have been studied in laboratory animals and appear to show little reproductive or developmental toxicity Nieuwenhuijsen et al. 2000a). In addition, recent studies found no association between swimming and excess risk of various birth outcomes (Klotz and Pyrch 1999; Nieuwenhuijsen et al. 2002; Waller et al. 1998), even though the potential for THM exposure and uptake during swimming may be high (Chu and Nieuwenhuijsen 2002; Whitaker et al. 2003a). Nevertheless, THMs may be acting as a surrogate measure for other chlorination by-products (e.g., the HAAs). These show some capacity for developmental effects but only at very high doses (Nieuwenhuijsen et al. 2000a). To date, they have not been a focus for epidemiologic investigation because of the lack of routinely collected data on these compounds. Klotz and Pyrch (1999) found only small associations of HAAs and haloacetonitriles with neural tube defects, but study power was low and CIs were wide. Other chlorination by-products, including the highly mutagenic chlorinated furanone MX, show little or no reproductive or developmental toxicity except at very high doses (International Programme on Chemical Safety 2000). However, not all potential chlorination byproducts have been identified yet. In addition, not all those that are known have been comprehensively studied for reproductive and developmental toxicity, and in most cases the substances have been studied separately rather than as a mixture, to which humans are generally exposed.

In summary, our findings overall suggest a significant association of stillbirths with maternal residence in high-TTHM exposure areas. Further work is needed to examine cause-specific stillbirths and effects of other DBPs and to explore the possibility of residual confounding at the individual level to help differentiate between alternative (non-causal) explanations and those that may be due to the water supply. The finding of significant heterogeneity between regions in the effect of TTHMs on risk of low and very low birth weight also deserves further study to understand better the reasons for heterogeneity, including possible differences in composition of other DBPs between water regions. Although the limited data from laboratory and epidemiologic studies do not so far indicate a causal association between exposure to THMs and stillbirth in humans, it would seem appropriate that water suppliers continue to follow the current policy of reducing THMs and other DBPs in public water supplies, as far as is consistent with maintaining effective control against waterborne microbiologic disease.

## Figures and Tables

**Table 1:** Descriptive data for the study population, by water region and TTHM category, 1992–1998.

		TTHM (μg/L)		Stillbirths		Low birth weight		Very low birth weight		
Water region/TTHM category	Carstairs score[mean (5th, 95th percentile)]	Mean (5th, 95th percentile)	No.*^a^*	Prevalence(95% CI)	No.*^a^*	Prevalence(95% CI)	No.*^a^*	Prevalence(95% CI)	No.*^a^*	Birth weight[g; mean (5th, 95th percentile)]
Northumbrian										
Low	1.68 (–2.83, 5.55)	18.0 (8.3, 29.0)	6	4.8 (1.0–8.6)	80	64.1 (50.5–77.7)	12	9.6 (4.2–15.0)	1,248	3,350 (2,380, 4,200)
Medium	1.53 (–3.30, 6.88)	48.1 (34.2, 58.9)	58	5.7 (4.2–7.1)	638	62.9 (58.2–67.6)	114	11.2 (9.2–13.3)	10,142	3,346 (2,410, 4,220)
High	1.54 (–3.62, 7.82)	71.5 (61.0, 88.2)	47	5.1 (3.7–6.6)	607	67.0 (61.8–72.1)	93	10.3 (8.2–12.3)	9,062	3,337 (2,380, 4,200)
Overall	1.54 (–3.45, 7.25)	56.6 (27.0, 81.1)	111	5.4 (4.4–6.4)	1,325	64.8 (61.4–68.2)	219	10.7 (9.3–12.1)	20,452	3,342 (2,390, 4,210)
United Utilities										
Low	−0.13 (–3.88, 5.82)	19.2 (6.4, 29.4)	192	4.3 (3.7–5.0)	2,665	50.6 (48.7–52.5)	405	7.7 (6.9–8.4)	52,662	3,396 (2,490, 4,260)
Medium	0.88 (–3.58, 7.58)	46.0 (32.6, 58.5)	1,194	5.3 (5.0–5.6)	15,882	59.9 (59.0–60.8)	2,336	8.8 (8.5–9.2)	265,030	3,356 (2,410, 4,220)
High	1.90 (–3.36, 8.21)	71.9 (60.8, 88.9)	824	5.8 (5.4–6.2)	10,197	68.0 (66.7–69.3)	1,521	10.1 (9.6–10.7)	149,905	3,326 (2,360, 4,200)
Overall	1.12 (–3.57, 7.71)	52.0 (19.0, 81.1)	2,210	5.4 (5.1–5.6)	28,744	61.5 (60.8–62.2)	4,262	9.1 (8.8–9.4)	467,597	3,351 (2,409, 4,220)
Severn Trent										
Low	0.54 (–3.54, 6.84)	11.2 (2.2, 28.9)	920	5.1 (4.7–5.4)	11,401	63.5 (62.4–64.6)	1,786	9.9 (9.5–10.4)	179,605	3,343 (2,390, 4,220)
Medium	0.86 (–3.57, 7.99)	44.0 (31.3, 57.8)	1,233	5.3 (5.0–5.6)	14,845	64.4 (63.4–65.4)	2,290	9.9 (9.5–10.3)	230,653	3,331 (2,381, 4,203)
High	0.26 (–3.61, 6.13)	70.7 (60.7, 88.6)	378	5.2 (4.7–5.7)	4,326	60.9 (59.2–62.7)	610	8.6 (7.9–9.3)	70,997	3,344 (2,410, 4,220)
Overall	0.65 (–3.57, 7.41)	35.8 (2.8, 72.5)	2,531	5.2 (5.0–5.4)	30,572	63.5 (62.8–64.2)	4,686	9.7 (9.5–10.0)	481,255	3,337 (2,399, 4,220)

Data are mean Carstairs scores, TTHM (μg/L) concentrations, prevalence and 95% CIs of stillbirths per 1,000 total births, low and very low birth weight per 1,000 live births, and mean birth weight (g). Prevalence of stillbirths, mean Carstairs score (the lower the score, the more affluent the area), and mean TTHM were based on total births for Northumbrian, 1997; United Utilities, 1993–1997; and Severn Trent, 1993–1998. Birth weight variables were based on live births for Northumbrian, 1997; United Utilities, 1992–1997; Severn Trent, 1993–1998. TTHM was categorized as follows: low, < 30 μg/L; medium, 30–59 μg/L; and high, ≥60 μg/L.

a Number of stillbirths, low-birth-weight births, and very-low-birth-weight births, and for birth weight, number of live births.

**Table 2:** Adjusted ORs*^a^* (95% CIs) for stillbirths and low and very low birth weight by TTHM category and by water region and overall, 1992–1998.

Water region/TTHM category	Stillbirths*^b^*	Low birth weight	Very low birth weight
Northumbrian			
Low	1.00	1.00	1.00
Medium	1.19 (0.51–2.75)	1.02 (0.80–1.30)	1.20 (0.66–2.18)
High	1.09 (0.46–2.55)	1.11 (0.87–1.41)	1.11 (0.61–2.03)
United Utilities			
Low	1.00	1.00	1.00
Medium	1.16 (1.00–1.35)	1.11 (1.07–1.16)	1.09 (0.98–1.21)
High	1.21 (1.03–1.42)	1.19 (1.14–1.24)	1.20 (1.07–1.34)
Severn Trent			
Low	1.00	1.00	1.00
Medium	1.03 (0.95–1.13)	1.00 (0.98–1.03)	1.00 (0.94–1.06)
High	1.04 (0.93–1.18)	0.98 (0.95–1.02)	0.90 (0.82–0.99)
Overall summary*^c^*			
Low	1.00	1.00	1.00
Medium	1.06 (0.99–1.15)	1.05 (0.96–1.15)	1.03 (0.96–1.10)
High	1.11 (1.00–1.23)	1.09 (0.93–1.27)	1.05 (0.82–1.34)

a ORs for stillbirths are adjusted for maternal age and Carstairs quintile and based on total births for Northumbrian, 1997; United Utilities, 1993–1997; and Severn Trent, 1993–1998. Regression analysis for birth weight variables is based on live births for Northumbrian, 1997; United Utilities, 1992–1997; and Severn Trent, 1993–1998. ORs for low birth weight are adjusted for maternal age, Carstairs quintile, sex of baby, and year of study (year was omitted in the case of Northumbrian). ORs for very low birth weight are adjusted for maternal age, Carstairs quintile, and year of study (year was omitted in the case of Northumbrian).

b Overall summary estimates for stillbirths are shown from the random-effects model for consistency with the birth weight estimates even though statistically significant heterogeneity between water regions was not found. However, results from a fixed-effects model were virtually identical.

c Overall summary estimates were obtained from random-effects model combining the region-specific exposure ORs allowing for heterogeneity between regions. *p*-Values for tests for heterogeneity (medium:low, high:low) from random-effects model were as follows: stillbirths (0.449, 0.339), low birth weight (0.000, 0.000), and very low birth weight (0.322, 0.001).
